# Preoperative prediction of lymph node metastasis in pancreatic ductal adenocarcinoma using MRI-derived whole-tumor ADC histogram analysis

**DOI:** 10.3389/fmed.2025.1736306

**Published:** 2026-01-12

**Authors:** Kun Chen, Yong Mei, Maoli Xu, Shihan Shi, Geya Tang, Yu-Feng Wang, Min Li, Yujie Wang, Xingzheng Pan, Zhibing Ruan

**Affiliations:** 1 Department of Radiology, The Affiliated Hospital of Guizhou Medical University, Guiyang, China; 2 Department of Hepatobiliary Surgery, The Affiliated Hospital of Guizhou Medical University, Guiyang, China; 3 Guizhou Medical University, Guiyang, China; 4 Institute of Medical Physics, School of Physics, University of Sydney, Sydney, NSW, Australia; 5 Department of Physiology, School of Medical Science, University of Auckland, Auckland, New Zealand

**Keywords:** apparent diffusion coefficient, histogram, lymph node metastasis, magnetic resonance imaging, pancreatic ductal

## Abstract

**Objective:**

To evaluate the predictive value of whole-tumor apparent diffusion coefficient (ADC) histogram parameters derived from MRI for assessing lymph node metastasis (LNM) in pancreatic ductal adenocarcinoma (PDAC).

**Materials and methods:**

Preoperative MRI and clinical data from 53 patients with pathologically confirmed PDAC were retrospectively analyzed. Patients were divided into two groups: LNM (*n* = 29) and non-LNM (NLNM, *n* = 24). ADC maps were generated from diffusion-weighted images acquired on a 3.0 T MRI scanner. Whole-tumor regions of interest were delineated in FireVoxel software to extract the full-volume ADC histogram parameters. A predictive model was developed and assessed using ROC analysis.

**Results:**

All ADC histogram parameters except the coefficient of variation and kurtosis showed significant differences between LNM and NLNM groups (*p* < 0.05); the first-order ADC values of LNM were significantly lower than those of NLNM. Baseline clinical characteristics (age, sex, clinical symptoms, CA19-9 levels) and conventional MRI features (size and volume) did not differ significantly. The multi-parameter model, based on select ADC-derived metrics, achieved an AUC of 0.865, with 86.2% sensitivity and 75.0% specificity.

**Conclusion:**

Whole-tumor ADC histogram analysis provides a non-invasive and quantitative tool for preoperative prediction of lymph node metastasis in PDAC. The integrated multiparametric model demonstrates superior diagnostic performance compared with single-parameter analysis.

## Introduction

Pancreatic ductal adenocarcinoma (PDAC) is a highly lethal malignant tumor characterized by insidious onset, propensity for local vascular invasion and metastasis, chemotherapy resistance, and an overall poor prognosis, with a 5-year survival rate of approximately 10% (1, 2). Surgical resection remains the only potential curative opportunity for PDAC. However, most patients present with advanced-stage disease at diagnosis and are therefore ineligible for surgery (3). Previous studies have identified lymph node metastasis (LNM) as a major adverse prognostic factor in PDAC (4, 5). Although endoscopic ultrasound-guided fine needle aspiration (EUS-FNA) is currently the gold standard for preoperative LNM assessment, it has several limitations, including its invasiveness, complex procedures, risk of complications, and susceptibility to interference from lesion location and surrounding anatomical structures (6, 7). Consequently, developing a non-invasive, comprehensive, and accurate preoperative technique to evaluate LNM status in PDAC is essential for improving patient stratification and guiding precision treatment planning.

Previous studies employing computer tomography (CT) imaging and artificial intelligence approaches have shown promising results in predicting LNM in PDAC, achieving satisfactory diagnostic performance (8, 9). However, while CT provides high-resolution anatomical detail and allows accurate measurement of tumor size and morphology, it primarily reflects macroscopic features. The microstructural alterations underlying PDAC progression, such as variation in cellular density and fibrosis, are often not captured by CT (10). In contrast, multiparametric magnetic resonance imaging (MRI) offers superior soft tissue contrast and the ability to characterize tissue at a microstructural and functional level, thereby becoming an emerging tool in pancreatic imaging (11–13). Diffusion-weight imaging (DWI) is an MRI technique in which the signal intensity reflects the random motion of water molecules within tissues (14). The apparent diffusion coefficient (ADC), a quantitative parameter derived from DWI acquired with multiple *b*-values, has been increasingly applied in the diagnosis and characterization of PDAC (15, 16). Previous studies have demonstrated significant associations between ADC values and both metastatic potential and prognosis in PDAC (17, 18). Moreover, machine learning models incorporating preoperative ADC values and clinical features have shown promise in predicting postoperative survival and recurrence risk in patients with PDAC (8).

These studies, however, were based on analyzing a 2D slice with the largest cross-section due to the location and size of the pancreas (8, 9). The 2D approach is insufficient to capture the full content of intratumor heterogeneity within a three-dimensional (3D) volume. In contrast, whole-tumor ADC histogram analysis provides a voxel-wise, multi-dimensional assessment that decodes the distribution of diffusion values across the entire volume of interest (VOI). Analyzing the whole tumor offers a more comprehensive illustration of the intratumoral microenvironment, which may be underestimated when using single-slice ADC measurements.

Building on these advances, the present study aims to investigate whether whole-tumor ADC histogram parameters derived from DWI could serve as non-invasive imaging biomarkers for predicting LNM in PDCA. Furthermore, we constructed and evaluated a multi-parameter predictive model based on these histogram-derived metrics to assess its diagnostic performance in differentiating LNM from NLNM cases. The findings from our study could provide imaging-based evidence supporting the feasibility of histogram-derived ADC metrics as quantitative indicators of tumor aggressiveness in PDAC, which may serve as guidelines for personalized treatment planning.

## Materials and methods

### Patients

**FIGURE 1:**
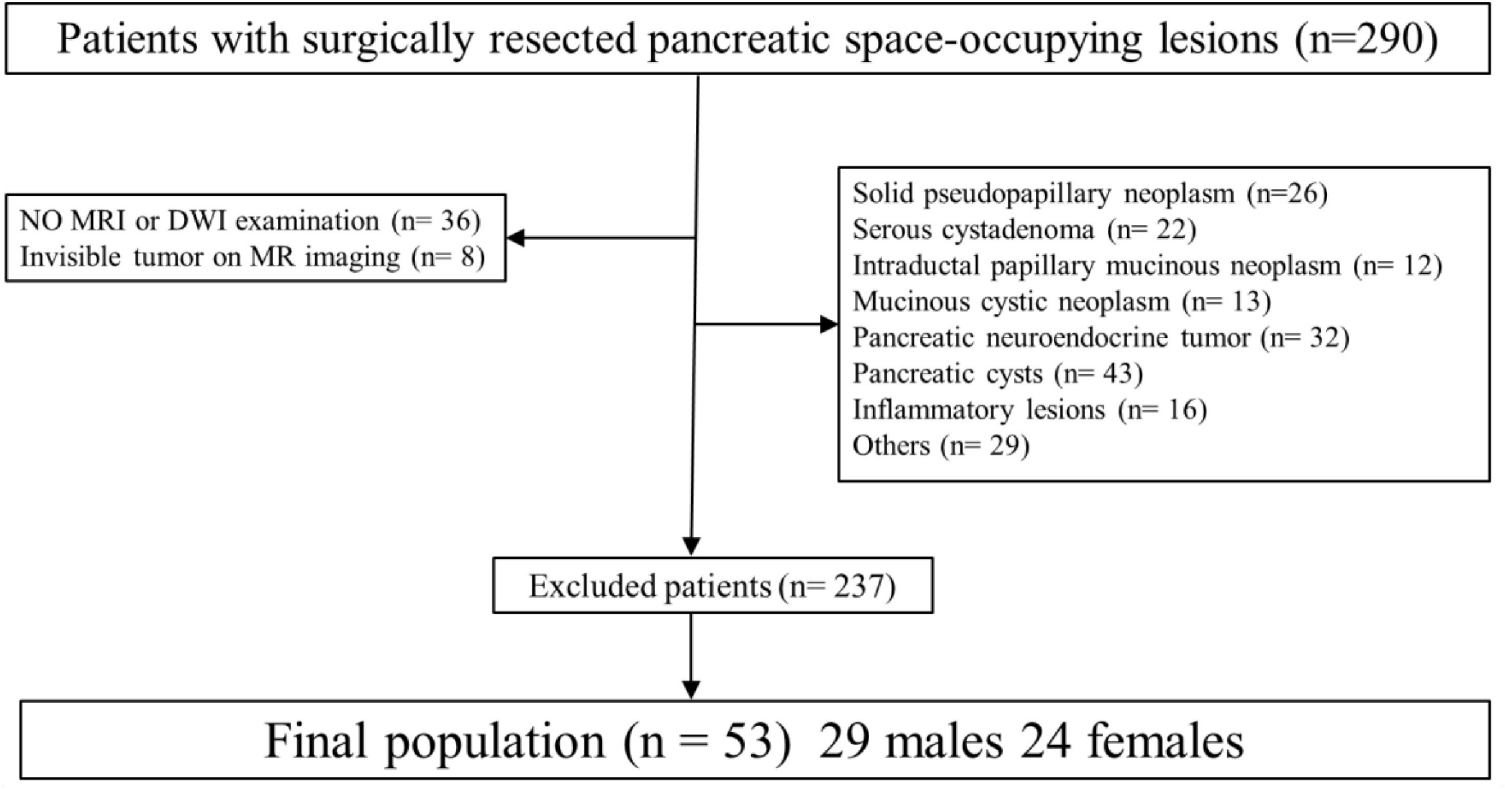
Flow chart of included and excluded patients.

This study was conducted in accordance with the principles of the Declaration of Helsinki and was approved by the Ethics Committee of the Affiliated Hospital of Guizhou Medical University (Approval No. 2024-512). The requirement for informed consent was waived due to the retrospective design. Clinical, imaging, and pathological data from patients with surgically confirmed pancreatic ductal adenocarcinoma (PDAC) treated between March 2022 and March 2025 were retrospectively reviewed. Inclusion criteria were as follows: (1) pathologically confirmed PDAC after surgical resection, stratified into lymph node metastasis (LNM) and non-lymph node metastasis (NLNM) groups; (2) preoperative MRI, including diffusion-weighted imaging (DWI), performed within 2 weeks before surgery; (3) no history of neoadjuvant chemoradiotherapy or other anti-tumor therapy before MRI; and (4) complete clinical and imaging records available. Exclusion criteria were as follows: (1) concurrent primary malignancy at another site; (2) inadequate image quality for quantitative analysis; and (3) tumor diameter ≤ 5 mm, precluding reliable measurement (Figure 1). After screening, a total of 53 patients with PDAC met the inclusion criteria and were enrolled in the study. Demographic and clinical data, including age, sex, presenting symptoms, and serum CA19-9 levels, were recorded for all patients (Table 1).

**TABLE 1:** MRI parameters.

Sequences	T1WI	T2WI	DWI
TR (ms)	1.15	2,105	2,900
TE (ms)	2.3	100	64
FOV (mm^2^)	360 × 309	380 × 380	380 × 380
Matrix	256 × 171	252 × 252	256 × 256
Slice thickness (mm)	6.0	6.0	6.0
Intersection gap (mm)	1.0	1.0	4.0

### MRI acquisition and imaging parameters

The MRI scans were acquired as part of routine standard of care. All examinations were conducted on a 3.0 T MRI system (Elition, Philips Healthcare, the Netherlands) equipped with a dedicated 32-channel phased-array abdominal coil. Prior to imaging, all patients fasted for 6∼8 h, which helps to maintain gallbladder distension and reduce gastrointestinal motion, thereby decreasing artifacts from the stomach and duodenum and improving pancreatic imaging quality. Patients underwent standardized breath-holding and respiratory training under the supervision of experienced radiographers. Scans were acquired in the supine position, with the imaging center positioned approximately 2∼3 cm inferior to the xiphoid process. Radiographers maintained iterative communication with patients to ensure comfort throughout the procedure, and short rest intervals were provided between sequences to minimize fatigue. The imaging protocol included T1-weighted imaging (T1WI), T2-weighted imaging (T2WI), and DWI sequences with b-values of 0 and 800 s/mm^2^. Detailed imaging parameters are summarized in Table 1. The total acquisition time was approximately 8–10 min if no repetition was needed.

### Image analysis

#### Tumor morphology analysis

Conventional imaging features, including tumor location, morphology, margin characteristics, pancreatic duct dilatation, parenchymal atrophy, and vascular invasion, were recorded for each patient. In brief, anatomical images obtained from T1WI and T2WI MRI were reviewed to determine tumor geometry. Tumor location was classified as involving the pancreatic head/neck, uncinate process, body, or tail. Morphology was categorized as regular or irregular, and margins were described as well-defined or poorly-defined. Additional features, such as pancreatic duct dilatation, parenchymal atrophy, and vascular invasion, were also documented. These imaging features were qualitatively evaluated by experienced radiographers. The tumor size, including the long- and short-axis diameters, was manually measured by radiographers using an in-house workstation.

#### ADC histogram analysis

ADC maps were analyzed using the freely available software package FireVoxel, build 473, accessed from https://firevoxel.org/ (19). The volumetric ADC datasets were imported into the software, and ROIs were manually delineated on each axial slice in which the tumor was clearly visualized. Corresponding anatomical images (T1WI and T2WI) were used as a reference to verify the accuracy of the manual segmentation on the ADC maps. To comprehensively reflect the overall heterogeneity of the tumor, the entire tumor volume was delineated as a whole to obtain histogram information, thus including cystic and necrotic components of the tumor. Manual delineation was employed for tumor contouring without using semi-automated/automated delineation tools. Following segmentation, voxel-wise ADC values within the ROIs were extracted to generate histograms, while the tumor volumes were calculated by the sum of voxels within the ROI. Multiple first-order statistical features were derived from the ADC histogram, including ADC-min, ADC-max, ADC-mean, standard deviation, coefficient of variation, skewness, kurtosis, entropy, and percentile values (ADC_1, 5, 10, 25, 50, 75, 90, 95, and 99%).

#### Intra/inter observer agreements

To minimize inter-observer bias, regions of interest (ROIs) were delineated independently by two radiologists with 4 and 20 years of experience, respectively. Both were blind to all clinical information. Each ROI encompassed the entire tumor while avoiding partial-volume effects by maintaining a 1∼2 mm margin within the tumor boundary. Any inter-observer discrepancies were resolved by consensus. For reproducibility assessment, inter-observer variability was evaluated by comparing the independently delineated ROIs of the two radiologists, while intra-observer variability was assessed by having one radiologist repeat the ROI delineation after a 4-week interval.

### Pathological evaluation

All surgical specimens were processed according to standardized pathological protocols, including fixation, paraffin embedding, dehydration, sectioning, haematoxylin-eosin (HE) staining, and slide preparation. The final study cohort consisted of 53 patients, divided into two groups: those with lymph node metastasis (LNM, *n* = 29) and those without lymph node metastasis (NLNM, *n* = 24). The determination of lymph node metastasis status was obtained from the pathology reporting system. All histopathological sections were independently reviewed by two gastrointestinal pathologists with 7 and 15 years of experience, respectively, who were blinded to all imaging and clinical data. Any discrepancies in interpretation were resolved through joint consensus review.

### Statistical analysis

All statistical analyses were performed using SPSS Statistics version 26.0 (IBM Corp., Armonk, NY, United States). Data normality was assessed using the Shapiro–Wilk test. Variables with a normal distribution were expressed as mean ± standard deviation and compared between groups using the independent samples *t*-test. Non-normally distributed variables were expressed as median (interquartile range) and compared using the Mann–Whitney U test. Categorical variables were presented as frequencies or percentages, and intergroup differences were evaluated using the χ^2^-test or Fisher’s exact test where appropriate.

Inter- and intra-observer agreement for quantitative parameters was assessed using the intra-class correlation coefficient (ICC); an ICC value greater than 0.75 was considered to indicate good reliability. When a good agreement was achieved, the mean of the parameters derived from the measurements of two observers was used for further analysis.

### Predictive model development

To further explore the clinical value of the DWI protocol, predictive models were constructed using ADC-derived histogram parameters. Logistic regression was employed for model fitting in SPSS. Each histogram parameter was first evaluated in an individual (single-parameter) model. Subsequently, a combined model incorporating multiple informative parameters from the histogram analysis was developed, so-called the multiparametric model. Diagnostic performance was evaluated by constructing receiver operating characteristic (ROC) curves and calculating the area under the curve (AUC) for each parameter. The optimal threshold value for each ROC curve was determined using the Youden index, which maximizes the sum of sensitivity and specificity. After that, the indices demonstrating relatively strong diagnostic performance (AUC > 75%) were selected to construct the multiparameter predictive models. The model construction and ROC analysis were done in SPSS. Comparisons between AUCs were performed using the DeLong test. A *p* < 0.05 was considered statistically significant for all analyses.

## Results

In this study, we aim to evaluate whether MRI-derived ADC metrics could predict LMN in PDAC patients. Data were retrospectively reviewed according to predefined selection criteria, and patients who met these criteria were included for analysis (Figure 1). We first present representative image data and histology results from one patient with LNM and one without (NLNM), followed by the group-level quantitative comparison. Finally, we describe the development and validation of the multiparameter predictive model.

### Demonstration of representative LNM and NLNM patient

**FIGURE 2:**
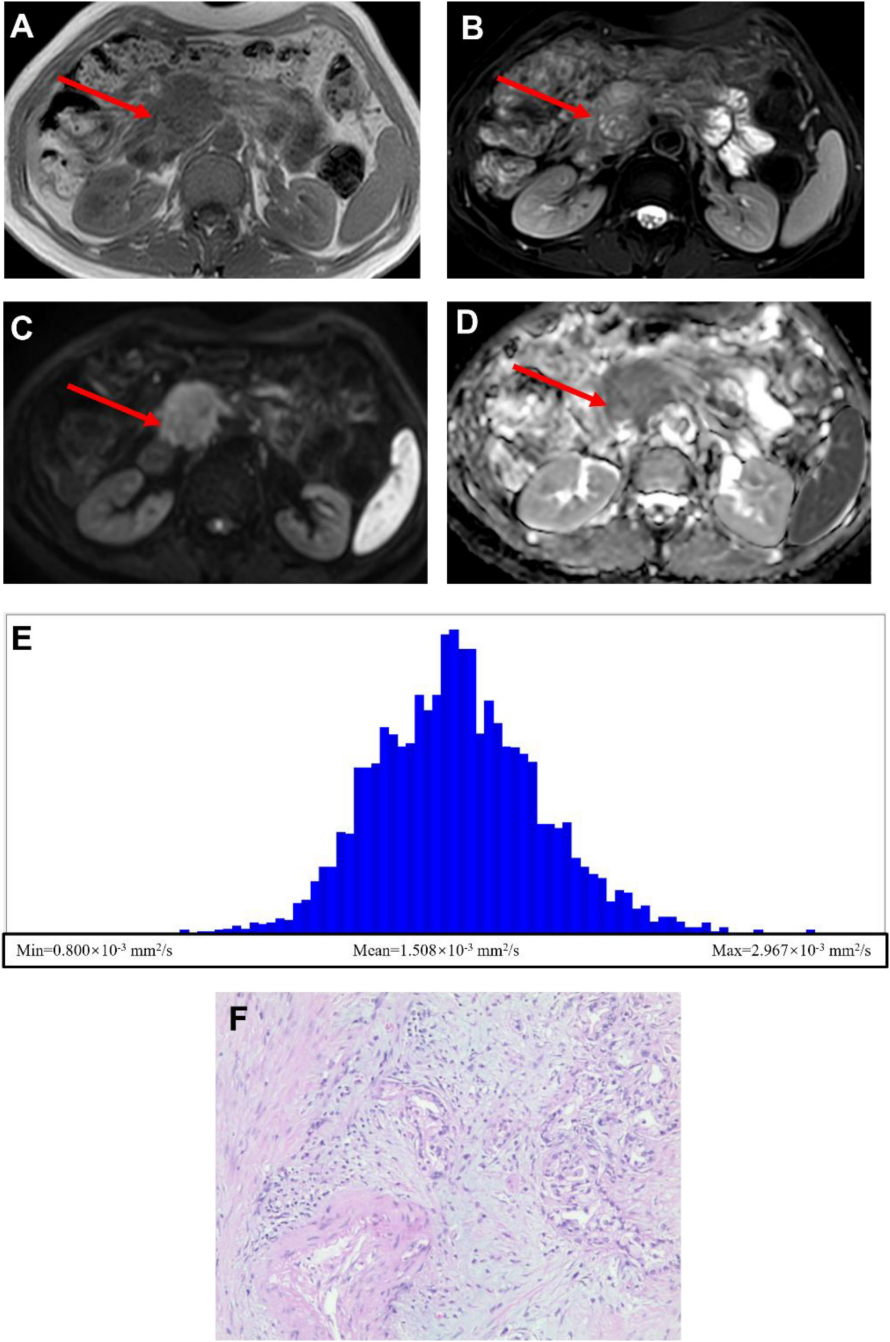
A 72-year-old female patient with pancreatic ductal adenocarcinoma and lymph node metastasis (LNM). **(A)** Axial T1-weighted image showing a mass-like lesion with isointense signal intensity in the pancreatic head. **(B)** Axial T2-weighted image demonstrating heterogeneous signal intensity within the lesion, predominantly mildly hyperintense. **(C)** Diffusion-weighted image (*b* = 800 s/mm^2^) showing hyperintensity in the lesion. **(D)** Calculated ADC map in which the lesion appears to have a higher diffusion rate. **(E)** Whole-tumor ADC histogram analysis showing the following quantitative parameters: ADC-min = 0.800 × 10^–3^ mm^2^/s, ADC-mean = 1.508 × 10^–3^ mm^2^/s, and ADC-max = 2.967 × 10^–3^ mm^2^/s. **(F)** Histopathological section confirming pancreatic ductal adenocarcinoma with LNM (H&E × 100).

**FIGURE 3:**
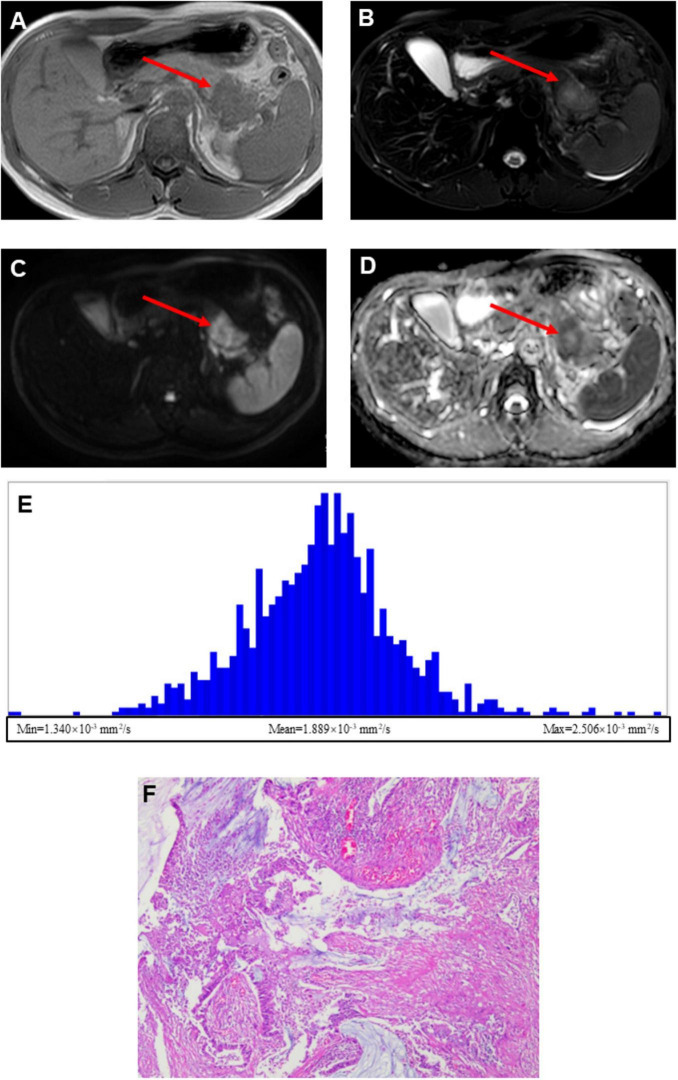
A 35-year-old male patient with pancreatic ductal adenocarcinoma and pathologically confirmed absence of lymph node metastasis (NLNM). **(A)** Axial T1-weighted image showing a mass-like lesion with isointense signal intensity in the pancreatic tail. **(B)** Axial T2-weighted image demonstrating heterogeneous signal intensity, predominantly mild hyperintensity at the periphery with a small focal area of prominent hyperintensity in the central region. **(C)** Diffusion-weighted image (*b* = 800 s/mm^2^) showing marked heterogeneous hyperintensity throughout the lesion. **(D)** Calculated ADC map demonstrating mild hyperintensity in the central portion with hypointense signals along the peripheral margins. **(F)** Whole-tumor ADC histogram analysis showing the following quantitative parameters: ADC-min = 1.340 × 10^–3^ mm^2^/s, ADC-mean = 1.889 × 10^–3^ mm^2^/s, and ADC-max = 2.506 × 10^–3^ mm^2^/s. **(E)** Histopathological section (H&E, ×100) confirming pancreatic ductal adenocarcinoma without lymph node metastasis.

Figure 2 presents the data from the PDAC with LNM patient, whereas Figure 3 presents the data from the NLNM patient. The lesions were clearly visualized on both T1-weighted (Figures 2A, 3A) and T2-weighted (Figures 2B, 3B) images, appearing distinct from the surrounding pancreatic parenchyma. Diffusion-weighted imaging (DWI) with a b-value of 800 s/mm^2^ (Figures 2C, 3C) demonstrated marked hyperintensity within the lesions, indicating restricted water diffusion. The calculated ADC maps derived from DWI (Figures 2D, 3D) showed areas of low signal intensity consistent with diffusion restriction. Subsequent whole-tumor histogram analysis of the ADC maps was performed using FireVoxel software (Figures 2E, 3E) to compute quantitative ADC metrics. The LNM case exhibited lower mean ADC values and a broader distribution range compared with the NLNM case, reflecting more restricted and heterogeneous diffusion characteristics. Finally, histopathological examination confirmed the diagnosis and lymph node status in both patients (Figures 2F, 3F).

### Demographics of patients and tumor characteristics

Among the 53 PDAC patients included in this study, 29 were diagnosed with LNM (aged 35–82 years, 60.17 ± 10.90 years), and 24 were in the NLNM group (aged 23–82 years, 58.46 ± 13.87 years). The clinical data and imaging features of the tumors of the LNM group and the NLNM group are shown in Table 2. There were no significant differences in gender, age, clinical symptoms (abdominal pain, jaundice), smoking, drinking, diabetes, hypertension, CA19-9, lesion location, morphology, long-axis and short-axis diameters, boundary, pancreatic duct dilatation or pancreatic parenchymal atrophy, and vascular invasion between the two groups (*p* > 0.05).

**TABLE 2:** Comparison of clinical baseline characteristics and conventional imaging features between the LNM and NLNM groups.

Baseline characteristics	LNM group (*n* = 29)	NLNM group(*n* = 24)	Statistic	*P-*value
Gender (Male/Female)	17/12	12/12	0.394^b^	0.530
Age (years)	60.17 ± 10.90	58.46 ± 13.87	0.504^a^	0.617
Abdominal pain (present/absent)	23/6	16/8	1.080^b^	0.299
Jaundice (present/absent)	13/16	9/15	0.290^b^	0.590
Weight loss (present/absent)	12/17	7/17	0.852	0.356
Smoking history (yes/no)	12/17	12/12	0.394^b^	0.530
Alcohol consumption (yes/no)	10/19	8/16	0.008^b^	0.930
Diabetes mellitus (present/absent)	8/21	4/20	0.894^b^	0.344
Hypertension (present/absent)	12/17	4/20	3.805^b^	0.051
CA19-9 (elevated/normal)	24/5	17/7	1.066^b^	0.302
Tumor location	20	18	0.236^b^	0.627
Head/uncinate				
Body/tail	9	6		
Morphology			0.082^b^	0.775
Regular	7	5		
Irregular	22	19		
Margin	1	2	0.029^b^	0.866
Well-defined				
Poorly-defined	28	22		
Long-axis diameter (mm)	29.07 ± 13.95	27.83 ± 10.91	0.353^a^	0.725
Short-axis diameter (mm)	21.72 ± 8.23	21.04 ± 6.87	0.323^a^	0.188
Volume (cm^3^)	10.409 ± 8.502	9.987 ± 6.999	0.846^a^	0.195
Pancreatic duct dilation/atrophy	17	17	0.852^b^	0.356
Present				
Absent	12	7		
Vascular invasion	7	7	0.171^b^	0.679
Present				
Absent	22	17		

^a^Data conforming to a normal distribution are presented as mean ± standard deviation; Intergroup comparisons were performed using the *t*-test.

^b^Categorical variables are expressed as proportions (%); Intergroup comparisons were analyzed using theχ^2^-test.

### Comparison of whole-tumor ADC histogram parameters between LNM and NLNM groups

Because manual ROI delineation was employed in this study, potential observer-related bias was taken into consideration. To minimize this effect and ensure analytical reliability, both intra- and inter-observer reproducibility of the ROI-based protocol were evaluated. The intraclass coefficients (ICC) were reported in Table 3. The whole-tumor ADC histogram parameters extracted from ROIs delineated by two radiographers demonstrated good inter-observer agreement (ICC > 0.75) (all *p* < 0.001) (Table 3).

**TABLE 3:** Consistency test results of each parameter measured by two observers.

Histogram parameters	Observer A	Observer B	ICC (95% *CI*)	*P*-value
ADC-min	1.142 ± 0.293	1.196 ± 0.303	0.864(0.776∼0.919)	<0.001
ADC-max	2.766 ± 0.536	2.694 ± 0.519	0.803(0.682∼0.882)	<0.001
ADC-mean	1.720 ± 0.306	1.687 ± 0.344	0.938(0.896∼0.964)	<0.001
SD	0.244(0.173,0.0.354)	0.258(0.164,0.339)	0.953(0.921∼0.973)	< 0.001
CV	0.146(0.107,0.191)	0.156(0.124,0.196)	0.890(0.816∼0.935)	<0.001
Skewness	0.699(0.335,1.001)	0.702(0.264,0.995)	0.919(0.863∼0.952)	<0.001
Kurtosis	1.073(0.234,2.404)	1.169(0.342,2.189)	0.988(0.979∼0.993)	<0.001
Entropy	3.949 ± 0.232	3.948 ± 0.264	0.876(0.794∼0.926)	<0.001
ADC-1%	1.254 ± 0.269	1.314 ± 0.274	0.912(0.853∼0.948)	<0.001
ADC-5%	1.352 ± 0.264	1.351 ± 0.275	0.977(0.961∼0.987)	<0.001
ADC-10%	1.418 ± 0.265	1.444 ± 0.264	0.947(0.909∼0.969)	<0.001
ADC-25%	1.535 ± 0.273	1.553 ± 0.267	0.993(0.988∼0.996)	<0.001
ADC-50%	1.696 ± 0.306	1.713 ± 0.307	0.993(0.998∼0.996)	<0.001
ADC-75%	1.878 ± 0.359	1.896 ± 0.359	0.995(0.991∼0.997)	<0.001
ADC-90%	2.067 ± 0.421	2.087 ± 0.418	0.996(0.992∼0.997)	<0.001
ADC-95%	2.192 ± 0.441	2.219 ± 0.443	0.996(0.993∼0.998)	<0.001
ADC-99%	2.517 ± 0.517	2.530 ± 0.517	0.995(0.992∼0.997)	<0.001

Data conforming to a normal distribution are presented as mean ± standard deviation (S ± SD), and intergroup comparisons were performed using the *t*-test. Data not conforming to a normal distribution are expressed as median (Q25, Q75), with intergroup comparisons analyzed using the Mann-Whitney U test. The coefficient of variation (CV), skewness, kurtosis, and entropy are dimensionless parameters. All other parameters are expressed in units of × 10^–3^ mm^2^/s. SD, standard deviation; CV, coefficient of variation; ICC, intra-class correlation coefficient.

All whole-tumor first-order statistical features from ADC maps were significantly lower in the LNM group than in the NLNM group (all *p* < 0.05) (Table 4). In addition, the skewness of the ADC histogram was significantly higher in the LNM group compared with the NLNM group (*p* = 0.022) (Table 4).

**TABLE 4:** Comparison of whole-tumor ADC histogram parameters between LNM and NLNM groups

Histogram parameters	LNM group (*N* = 29)	NLNM group (*N* = 24)	Statistic	*P*-value
ADC-min	1.064 ± 0.285	1.236 ± 0.280	−2.213^a^	0.031
ADC-max	2.627 ± 0.582	2.934 ± 0.429	−2.150^a^	0.036
ADC-mean	1.563 ± 0.218	1.910 ± 0.291	−4.946^a^	< 0.001
SD	0.187(0.161, 0.281)	0.278(0.220, 0.398)	−2.162^b^	0.031
CV	0.132(0.102, 0.195)	0.156(0.116, 0.190)	−0.733^b^	0.464
Skewness	0.812(0.642, 1.021)	0.484(0.157, 0.849)	2.287^b^	0.022
Kurtosis	1.170(0.572, 2.724)	0.132(0.102, 2.195)	1.894^b^	0.058
Entropy	3.888 ± 0.195	4.023 ± 0.255	−2.167^a^	0.035
ADC-1%	1.154 ± 0.253	1.373 ± 0.242	−3.198^a^	0.002
ADC-5%	1.244 ± 0.239	1.484 ± 0.236	−3.666^a^	0.001
ADC-10%	1.304 ± 0.232	1.554 ± 0.241	−3.841^a^	< 0.001
ADC-25%	1.406 ± 0.215	1.693 ± 0.254	−4.460^a^	< 0.001
ADC-50%	1.539 ± 0.217	1.887 ± 0.293	−4.958^a^	< 0.001
ADC-75%	1.698 ± 0.247	2.096 ± 0.357	−4.779^a^	< 0.001
ADC-90%	1.869 ± 0.3.8	2.306 ± 0.419	−4.365^a^	< 0.001
ADC-95%	1.997 ± 0.335	2.429 ± 0.443	−4.037^a^	< 0.001
ADC-99%	2.339 ± 0.490	2.722 ± 0.483	−2.854^a^	0.006^b^

^a^Data conforming to a normal distribution are presented as mean ± standard deviation (S ± SD), and intergroup comparisons were performed using the *t*-test.

^b^Data not conforming to a normal distribution are expressed as median (Q25, Q75), with intergroup comparisons analyzed using the Mann-Whitney U test. The coefficient of variation (CV), skewness, kurtosis, and entropy are dimensionless parameters. All other parameters are expressed in units of × 10^–3^ mm^2^/s. SD, standard deviation; CV, coefficient of variation.

### Development of a predictive model for PDAC lymph node metastasis based on ADC histogram parameters

Having established the reliability of the imaging analysis, our results demonstrated that the whole-tumor ADC histogram metrics differed significantly between the LNM and NLNM groups, reflecting discriminative diffusion features attributable to underlying pathological differences. The disease status of all cases was confirmed by histopathological examination, providing the ground truth for model development. Therefore, it is feasible to develop a predictive model using histogram-derived ADC parameters for the preoperative identification of lymph node metastasis, with the potential to complement or even reduce dependence on invasive biopsy procedures.

**FIGURE 4:**
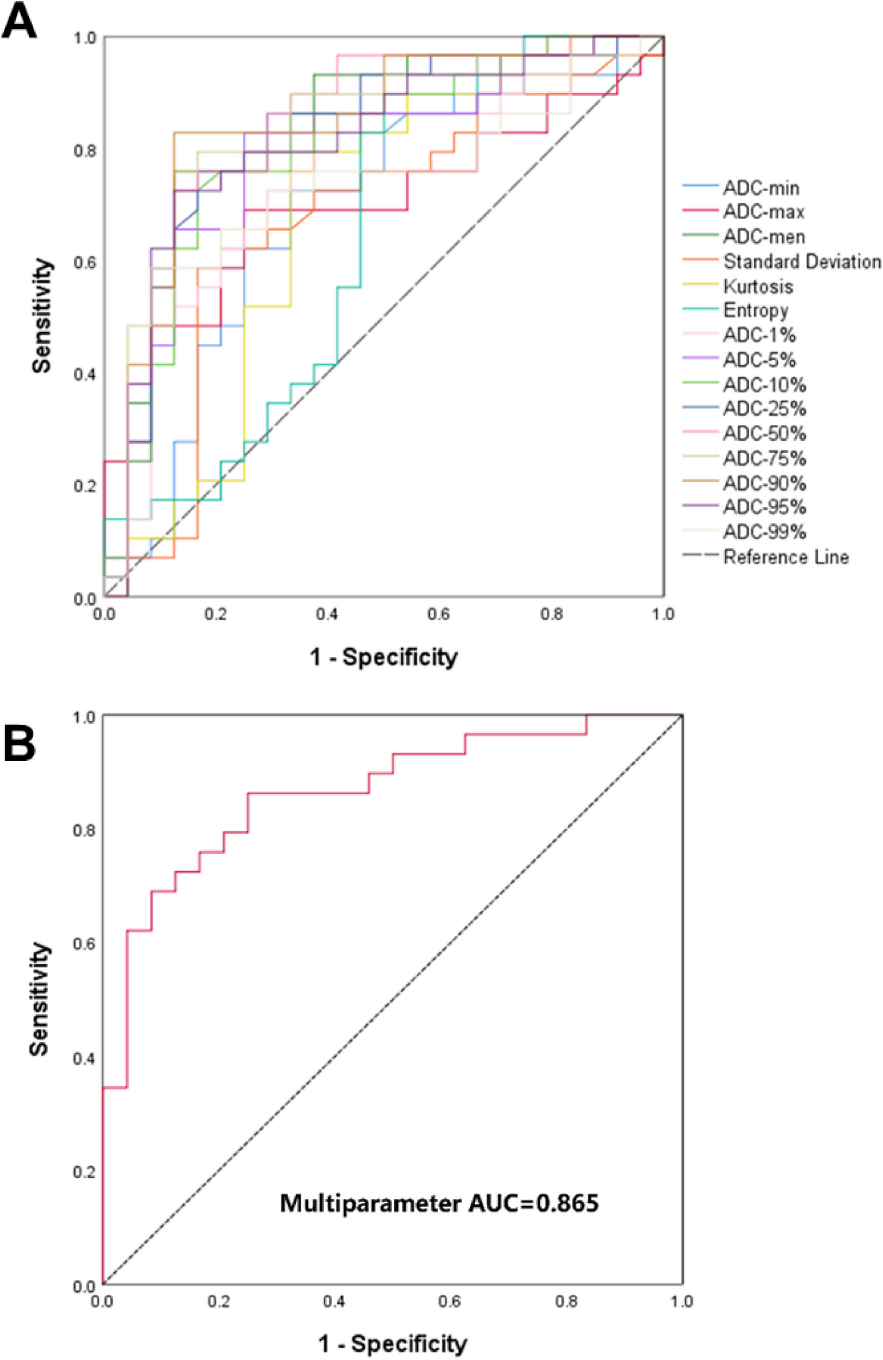
ROC curves for predicting lymph node metastasis in PDAC. **(A)** ROC curves of each ADC histogram-derived parameter, with the dashed line being the reference line. **(B)** ROC curve of the integrated multi-parameter prediction model introduced in our study.

The diagnostic performance of the model was first evaluated by ROC analysis to determine its sensitivity and specificity (Figure 4). The diagnostic performance of individual histogram-derived parameters, as quantified by the AUC, ranged from 0.634 to 0.845 across all metrics (Figure 4A and Table 4). Among these, ADC-50% demonstrated the best diagnostic performance, achieving an AUC of 0.845, with a sensitivity of 72.4% and a specificity of 87.5% at an optimal threshold of 1.666 × 10^–3^ mm^2^/s. Parameters that demonstrated relatively strong diagnostic performance—specifically ADC-mean, ADC-1%, ADC-5%, ADC-10%, ADC-25%, ADC-50%, ADC-75%, ADC-90%, and ADC-95% were selected to construct a multiparameter prediction model. These parameters were integrated to capture the important aspects of diffusion characteristics within the tumor, including both central tendency and percentile-based heterogeneity features.

Although the multiparameter model did not show a statistically significant improvement of prediction outcomes compared with individual parameter models (*p* > 0.05) (Table 5), except in the case of ADC-1% (*p* = 0.03), its numerically higher AUC (0.865), sensitivity (86.2%), and specificity (75%) indicate a more stable and reliable predictive performance (Figure 4C and Table 6).

**TABLE 5:** Results of Delong test comparing individual parameter models and the integrated multiparameter model.

Tested variable	Statistic	*P-*value
ADC-mean vs. multiparameter	−1.009	0.313
ADC-1% vs. multiparameter	−2.086	0.037
ADC-5% vs. multiparameter	−1.556	0.120
ADC-10% vs. multiparameter	−1.503	0.133
ADC-25% vs. multiparameter	−1.117	0.264
ADC-50% vs. multiparameter	−0.780	0.435
ADC-75% vs. multiparameter	−0.995	0.320
ADC-90% vs. multiparameter	−0.664	0.507
ADC-95% vs. multiparameter	−1.168	0.243

**TABLE 6:** ROC curve analysis of ADC histogram parameters and the integrated multiparameter diagnostic model.

Histogram parameters	AUC(95% CI)	Sensitivity/%	Specificity/%	Threshold ( × 10^–3^ mm^2^/s)	Youden index
ADC-min	0.690(0.542∼0.839)	72.4	66.7	1.252	0.391
ADC-max	0.697(0.554∼0.840)	65.5	75.0	2.692	0.405
ADC-mean	0.839(0.725∼0.953)	75.9	87.5	1.722	0.634
SD	0.674(0.521∼0.827)	58.6	83.3	0.199	0.419
Kurtosis	0.684(0.532∼0.836)	72.4	70.8	0.670	0.432
Entropy	0.634(0.475∼0.792)	86.2	50.0	4.027	0.362
ADC-1%	0.753(0.617∼0.888)	86.2	62.5	1.379	0.487
ADC-5%	0.790(0.613∼0.918)	82.8	75.0	1.421	0.578
ADC-10%	0.797(0.671∼0.922)	72.4	83.3	1.450	0.557
ADC-25%	0.825(0.707∼0.944)	75.9	83.3	1.550	0.592
ADC-50%	0.845(0.732∼0.958)	72.4	87.5	1.666	0.599
ADC-75%	0.838(0.723∼0.952)	75.9	87.5	1.854	0.634
ADC-90%	0.841(0.725∼0.956)	79.3	87.5	2.035	0.668
ADC-95%	0.813(0.692∼0.935)	72.4	87.5	2.087	0.599
ADC-99%	0.736(0.597∼0.875)	58.6	97.1	2.227	0.503
Multiparameter	0.865(0.767∼0.963)	86.2	75.0	–	0.612

## Discussion

Our study is the first to employ whole-tumor ADC histogram parameters for predicting LNM in PDAC patients. Our findings demonstrate that whole-tumor ADC histogram analysis has significant predictive value for LNM, with the multiparameter model achieving optimal diagnostic performance (AUC of 0.865, a sensitivity of 86.2%, and a specificity of 75.0%). Compared with conventional 2D analysis, which uses a slice containing the largest section of the pancreas, or mean-ADC analysis, histogram-based analysis on a volumetric basis provides the entire distribution of voxel-wise diffusion values, thereby reflecting the degree of inter-turned heterogeneity. This comprehensive assessment offers novel imaging-based biomarkers to support more precise clinical decision-making. On the other hand, this approach provides additional pathological insights into tumor microstructure and metastasis.

DWI plays an increasingly important role in the differential diagnosis of pancreatic adenocarcinomas (15, 16, 20, 21). In this study, we further employed FireVoxel, a quantitative medical image analysis software, to analyze the whole-tumor ADC values. FireVoxel offers voxel-wise analysis, automated processing, and extraction of histogram-based metrics to facilitate inter-group comparisons. This analytic approach has been applied in a range of oncological MRI studies, demonstrating its versatility and reliability across different tumor types and clinical applications (22–24). The present study revealed that the LNM group consistently exhibited lower ADC values than the NLNM group, a finding that is consistent with previous studies (17, 18). Although tumor size and volume did not differ significantly between the LNM and NLNM groups in our study (Table 3), the accompanying decrease in ADC values suggests that the metastatic mechanism in PDAC may not solely depend on macroscopic tumor burden but rather on cellular and microstructural characteristics that influence water diffusion behavior. A potential explanation is that tumors in the LNM group demonstrate higher cellular proliferation and aggressiveness, resulting in increased cellular density and reduced extracellular space. This pathological mechanism of lymph node metastasis aligns with other cancers, including cervical (25), rectal (26), and breast cancer (27).

In clinical practice, however, diagnosing lymph node metastasis based on ADC measurements of lymph nodes remains challenging. This difficulty arises primarily due to the frequent presence of multiple small lymph nodes in the peripancreatic region, which often complicates accurate ROI placement and leads to unreliable ADC measurements. To overcome these challenges, our study focused on ADC values derived from the primary tumor to enhance reliability. Future work may utilize advanced AI-based segmentation to better discriminate tumor tissues, reduce variability and improve ROI homogeneity, thereby enhancing reproducibility and minimizing observer bias.

Previous studies have reported that elevated CA19-9 levels may indicate the LNM in PDAC patients (28). However, in our study, no significant difference in CA19-9 levels was observed between the LNM and NLNM groups (Table 3), suggesting that the predictive role of serum tumor markers remains uncertain. The absence of a significant difference in CA19-9 levels between the LNM and NLNM groups may be related to confounding factors such as biliary obstruction, hepatic dysfunction, or limited cohort size (29, 30). In contrast, our study focused on using ADC values derived from the primary tumor as an imaging-based biomarker to infer the biological behavior.

DWI is a widely used imaging technique, and the derived ADC values provide meaningful quantitative information related to tumor cellularity and proliferative behavior. Building on this clinically accessible sequence, our study investigated whether ADC histogram parameters could assist in the preoperative assessment of lymph node status, and the results showed reasonable diagnostic performance. In practice, such an imaging-based, non-invasive approach may complement existing methods and, in selected patients, help reduce reliance on invasive procedures such as endoscopic ultrasound-guided biopsies, particularly when these are inconclusive or technically challenging. For instance, for patients identified with lymph node metastasis preoperatively, surgeons can exercise heightened vigilance during lymph node dissection, paying closer attention to the nodal basins, which would substantially support clinical decision-making and benefit patient outcomes. Although the multiparameter diagnostic model incorporating several ADC histogram metrics demonstrated numerically improved performance, statistical comparison using the DeLong test showed no significant difference compared with individual parameters, such as mean ADC. Nevertheless, the combined model provided higher AUC, sensitivity, and specificity, suggesting greater robustness and stability. Therefore, in routine clinical practice, mean ADC can serve as a practical and initial screening for predicting lymph node metastasis in PDAC, while a multiparameter model may offer additional value in complex cases where tumor heterogeneity is more pronounced.

## Limitations

One of the limitations of this study is a relatively small sample size, predominantly due to the low incidence and high aggressiveness associated with PDAC. Because most patients present with advanced or unresectable disease, the number of eligible surgical cases available for imaging analysis was limited, thereby reducing the statistical power of this study. Moreover, the retrospective design from a single institution lacks an external validation cohort, which may introduce selection bias and limit the generalizability of the results. Future studies may consider employing multi-center datasets with larger sample sizes to further validate the findings and refine the proposed predictive model. Finally, the present analysis was restricted to ADC histograms derived from DWI at a single b-value (800 s/mm^2^). Multi-b-value acquisitions, as well as diffusion kurtosis imaging (31, 32) and intravoxel incoherent motion (IVIM) modeling (12), could be explored to capture additional diffusion characteristics and provide a more comprehensive assessment of tumor microstructure, although these techniques require longer acquisition time that may be practically hard for PDAC patients.

## Conclusion

In conclusion, whole-tumor ADC histogram parameters serve as a quantitative technique for predicting LNM in PDAC patients. The multiparameter model demonstrates superior predictive efficacy compared to single-parameter approaches, offering a non-invasive and accurate imaging-based tool for preoperative assessment of nodal involvement. This advancement offers novel technical support for informed clinical decision-making in the management of PDAC. Future studies could conduct multicenter, large-scale analyses to further validate these findings and facilitate their integration into routine clinical practice, allowing for personalized therapeutic strategies.

## Data Availability

The raw data supporting the conclusions of this article will be made available by the authors, without undue reservation.
